# A Semipersistent Plant Virus Differentially Manipulates Feeding Behaviors of Different Sexes and Biotypes of Its Whitefly Vector

**DOI:** 10.3390/v9010004

**Published:** 2017-01-13

**Authors:** Shaohua Lu, Jingjing Li, Xueli Wang, Danyang Song, Rune Bai, Yan Shi, Qinsheng Gu, Yen-Wen Kuo, Bryce W. Falk, Fengming Yan

**Affiliations:** 1 College of Plant Protection, Henan Agricultural University, Zhengzhou 450002, China; shaohualu08@163.com (S.L.); lijingjing_319@163.com (J.L.); wxl2010@yeal.net (X.W.); songdanyang2@163.com (D.S.); yxbre@163.com (R.B.); shiyan00925@126.com (Y.S.); 2 Zhengzhou Fruit Research Institute, Chinese Academy of Agricultural Sciences, Zhengzhou 410100, China; guqinsheng@caas.cn; 3 Department of Plant Pathology, University of California, Davis, CA 95616-8600, USA; ywkuo@ucdavis.edu (Y.-W.K.); bwfalk@ucdavis.edu (B.W.F.)

**Keywords:** cucurbit chlorotic yellows virus, CCYV, *Bemisia tabaci*, electrical penetration graph, EPG, feeding behaviors

## Abstract

It is known that plant viruses can change the performance of their vectors. However, there have been no reports on whether or how a semipersistent plant virus manipulates the feeding behaviors of its whitefly vectors. Cucurbit chlorotic yellows virus (CCYV) (genus *Crinivirus*, family *Closteroviridae*) is an emergent plant virus in many Asian countries and is transmitted specifically by B and Q biotypes of tobacco whitefly, *Bemisia tabaci* (Gennadius), in a semipersistent manner. In the present study, we used electrical penetration graph (EPG) technique to investigate the effect of CCYV on the feeding behaviors of *B. tabaci*. The results showed that CCYV altered feeding behaviors of both biotypes and sexes of *B. tabaci* with different degrees. CCYV had stronger effects on feeding behaviors of Q biotype than those of B biotype, by increasing duration of phloem salivation and sap ingestion, and could differentially manipulate feeding behaviors of males and females in both biotype whiteflies, with more phloem ingestion in Q biotype males and more non-phloem probing in B biotype males than their respective females. With regard to feeding behaviors related to virus transmission, these results indicated that, when carrying CCYV, *B. tabaci* Q biotype plays more roles than B biotype, and males make greater contribution than females.

## 1. Introduction

Most plant viruses depend on vector insects to move from one host to another and over distantly-located regions [1,2,3,4]. The interaction between virus and vector is very specific and complex, and some studies reported that viruses could alter directly the physiology and behaviors of the vector to promote spread of the viruses [5,6,7,8,9,10,11]. The implications of the interactions among virus, vectors and plants in virus pandemics have attracted more and more attention in recent years [12,13].

*Cucurbit chlorotic yellows virus* (CCYV), as an emergent plant virus that belongs to genus *Crinivirus* in the family *Closteroviridae*, was firstly identified in melon (*Cucumis melo*) in Japan in 2004 [14], and now is spreading throughout China [15,16,17], Sudan [18], Lebanon [19], Iran [20], Greece [21] and Saudi Arabia [22]. CCYV is the single-stranded positive-sense RNA virus, composed of RNA1 (8607-nucleotide (nt)) and RNA2 (8041-nucleotide (nt)) [23]. CCYV can infect melon (*C. melo*), watermelon (*Citrullus lanatus*) and cucumber (*Cucumis sativus*) and other plant species, and cause chlorotic leaf spots and complete yellowing of leaves [17,23], resulting in severe yield and serious economic losses. CCYV is transmitted specifically by Q (“Mediterranean” putative species) and B (“Middle East-Asia Minor 1” putative species) biotypes of *Bemisia tabaci* (Gennadius) (Hemiptera: Aleyrodidae) in a semipersistent manner [23].

*B. tabaci* is a species complex consisting of at least 34 morphologically indistinguishable species [24]. The Q biotype and B biotype are the most widely distributed biotypes of the species [25]. B biotype was first detected in China in the mid-1990s. It replaced the indigenous whitefly species and became the dominant whitefly in both greenhouse and field crops [26]. This situation changed after Q biotype was found in China’s Yunnan Province in 2003 [27]. By 2009, Q biotype had displaced biotype B as the dominant whitefly in most locations in China [28]. *B. tabaci* is a destructive pest of vegetable and ornamental production worldwide [29], especially because of the role as a vector of plant viruses [30,31]. According to the International Committee on Taxonomy of Viruses, by 2013, *B. tabaci* can transmit 212 plant virus species in five genera, including *Begomovirus*, *Crinivirus*, *Ipomovirus*, *Carlavirus* and *Torradovirus* [32,33,34].

Electrical penetration graph (EPG) technique is a reliable tool in the studies on the feeding behaviors of piercing-sucking insects [35,36]. EPG waveforms representing the details of probing behaviors of insects in certain plant tissues [37] could help to interpret the interactions between plants and piercing sucking insects [38]. EPG has been extensively used in piercing-sucking insects’ research, such as the probing behaviors of insects on plants, the location of the feeding stimulants or antifeedants in plant tissues [39,40], and the roles insects play in transmission of plant viruses and other pathogens [4].

There have been many reports on alteration of physiology, molecular biology or feeding behaviors in *B. tabaci* by persistently transmitted plant viruses, mainly *Begomovirus*. But few or no studies are available on semipersistent viruses transmitted by *B. tabaci*. Here we decided to test whether semipersistent plant viruses have specific effects on their vectors’ performance. In the current work, we used the EPG technique to compare feeding behaviors of viruliferous or non-viruliferous B and Q biotypes and their males and females of *B. tabaci* on cotton (CCYV non-host) in an attempt to provide evidence for the studies of interaction mechanisms between CCYV and *B. tabaci* and lay the basis for effective monitoring and management of CCYV and its vectors. EPG variables of non-phloem and phloem phases indicated that feeding behaviors of *B. tabaci* B and Q biotypes changed greatly when carrying CCYV, with more phloem salivation and ingestion in both biotypes, stronger effects on Q biotype than on B biotype, and more changes in males than in females.

## 2. Materials and Methods

### 2.1. Plants

Plants of cotton (*Gossypium hirsutum* L. cv. Yinshan-1) (host plant for *B. tabaci*, but not for CCYV) and cucumber (*Cucumis sativus* L. cv. Lvjian-1) (host plant for both *B. tabaci* and CCYV) were grown in pots (d = 14.5 cm) in a greenhouse at 16:8 LD, 26 ± 1 °C and 75% ± 0.5% relative humidity. Cotton plants of 3 to 4 true-leaf stage were used in the experiments.

To obtain CCYV-infected plant cultures, cucumber plants at 1–2 true-leaf stage were inoculated with *Agrobacterium tumefaciens*-mediated CCYV clones [41]. Plants were then left to grow for 30 days, and infection status was determined by symptom of chlorotic leaf spots and yellowing, and subsequently confirmed by real-time RT-PCR. All plants were maintained in separate insect-proof cages under a greenhouse under the above conditions.

### 2.2. Laboratory Whitefly Populations

The whitefly populations of *B. tabaci* B and Q biotypes were reared on healthy tobacco (*Nicotiana tabacum* cv. Zhongyan-100) plants for many years in whitefly-proof screen cages (60 cm × 40 cm × 80 cm) under conditions as above, respectively. The purities of *B. tabaci* B and Q biotype populations were monitored every 4–5 generations by using the mitochondrial cytochrome oxidase I (*mtCOI*) genes [42,43].

### 2.3. Establishment of Non-Viruliferous and Viruliferous B. Tabaci Populations

We established non-viruliferous and viruliferous whitefly colonies by transferring ca. 600 adults of *B. tabaci* B and Q biotypes from above laboratory populations into each cage with two virus-free or CCYV-infected cucumber plants, respectively. Viruliferous and non-viruliferous whitefly colonies were maintained for 2 generations in a greenhouse under above conditions. Starting from the third generation, we randomly selected newly emerged whiteflies (2 to 5 day-old males and females) from each colony for use in the experiments.

Viruliferous status of whiteflies were examined using real-time RT-PCR. Primers were designed based on CCYV coat protein sequence (GenBank accession No. HM581658.1) using Primer Premier 5 software (Premier Biosoft International, Palo Alto, CA, USA). The forward primer (CCYV-F, 5′-GCGACCATCATCTACAGGCA-3′, nucleotide positions 548–567) and the reverse primer (CCYV-R, 5′-CCGACTTGTTCCTTTCAGAGC-3′, nucleotide positions 679–699) were used to generate a 430 base pair fragment.

Total RNA of the individual adult whitefly from CCYV-infected or non-infected plants was extracted using TRlzol reagent (Invitrogen, Carlsbad, CA, USA) following the manufacturer’s instructions. RNA concentration and purity were measured in a NanoDrop^TM^ spectrophotomer (Thermo Scientific, Wilmington, DE, USA) and stored at −20 °C for subsequent analysis. Total RNA (1 µg) from each sample was reverse transcribed to generate the first-strand cDNA using the PrimeScript^®^ RT reagent Kit (Takara, Dalian, China).

The cocktail for PCR amplification was a mixture of 20 µL containing 10 µL Premix Taq™ (Ex Taq™ Version 2.0 plus dye, Takara, Dalian, China), 0.5 µL (5 µM) of each primer (CCYV-F and CCYV-R), 1 µL of cDNA sample and 8 µL ddH_2_O. The PCR amplification conditions were: an initial denaturation phase of 2 min at 94 °C, followed by 35 cycles of amplification 94 °C for 30 s, 55 °C for 30 s and 72 °C for 30 s, and 10 min at 72 °C. PCR products (10 µL) were subjected to electrophoresis in a 1.5% agarose gel 1× TAE buffer (40 mM Tris-acetate, pH 8.3, 1 mM EDTA), and observed on a UV-transilluminator after ethidium bromide staining.

### 2.4. Electrical Penetration Graph Recording

A direct-current EPG (DC-EPG, Model Giga-4) system (Wageningen University, Wageningen, The Netherlands) was used to monitor the feeding behaviors of viruliferous and non-viruliferous whitefly adults on cotton plants. We used cotton plants in the experiments because cotton is the excellent host plant for whiteflies, but not the host of CCYV, to avoid virus effects on non-viruliferous whiteflies that could mask the direct effects of the virus on its vector. Whiteflies were immobilized on the ice pack, then we attached a gold wire (1.5 cm × 12.5 μm) to the pronotum of a whitefly using a drop of water-based silver glue. The wired whiteflies were starved for ca. 20 min before connected to the Giga-4 probe input and placed onto the lower surface of the first true leaf. Six hours of EPGs were continuously recorded for each replicate with a fresh adult and a new plant. All experiments were carried out in a quiet room at 26 ± 1 °C, at 75% ± 0.5% relative humidity and under 1000 lux artificial light. All the recoding experiments were finished in an electrically grounded Faraday cage to shield against external electrical noise.

The EPG signals were digitized with a DI-720-UL analogue-to-digital converter (Dataq Instruments, Akron, OH, USA), and the output was acquired and stored with Stylet+ for Windows software (Wageningen University, Wageningen, The Netherlands), and data were analyzed with this software after data conversion.

### 2.5. Data Analysis

Waveforms patterns were categorized as previously described [44,45,46]. We identified five distinct waveforms in this study: non-probing (np; non-probing behavior); pathway [C; intercellular stylet pathway, including, if occurred, waveforms F (presumed penetration difficulties) and G (xylem sap ingestion)]; potential drop (pd; puncturing into the tissue cells and tasting the cytoplasm); the phloem phase E1 (watery saliva secretion into a sieve element) and E2 (ingestion of sieve element sap). The time from the start to the end of each waveform was recorded and exported by using Stylet+ software.

We selected 16 EPG variables (8 non-phloem phase variables and 8 phloem phase variables) for analysis and comparison of feeding behaviors of different biotypes and different sexes of *B. tabaci* with or without CCVY. All statistical analyses were done with IBM SPSS Statistics 20.0 (IBM Corp., Armonk, NY, USA) and significant differences were tested at the 0.05 or 0.01 level. Prior to analysis, normality and homogeneity of variance were checked. Data were log10 transformed when it did not fit a normal distribution. Means of viruliferous and non-viruliferous whiteflies of the same sex and the same viruliferous situation were compared by Mann-Whitney test. Two-way analysis of variance (ANOVAs) was used to analyze the interaction effects of sex and whitefly viruliferous status (viruliferous and non-viruliferous whitefly). Multivariate analysis of variance was used to analyze the interaction effects of biotype (B and Q), sex (male and female), whitefly viruliferous status (non-viruliferous and viruliferous). Means were compared by least significant difference (Tukey’s) tests.

## 3. Results

A total of 166 successful EPG recordings were obtained on cotton plants, a non-host of CCYV, including 73 for *B. tabaci* B biotype whiteflies (37 non-viruliferous whiteflies with 18 replicates for males and 19 replicates for females, and 36 viruliferous with 17 replicates for males and 19 replicates for females), and 93 for Q biotype whiteflies (50 non-viruliferous with 20 replicates for males and 30 replicates for females, and 43 viruliferous with 22 replicates for males and 21 replicates for females).

### 3.1. Effects of CCYV on Non-Phloem Feeding Behaviors of B. tabaci B and Q Biotypes

**Figure 1:**
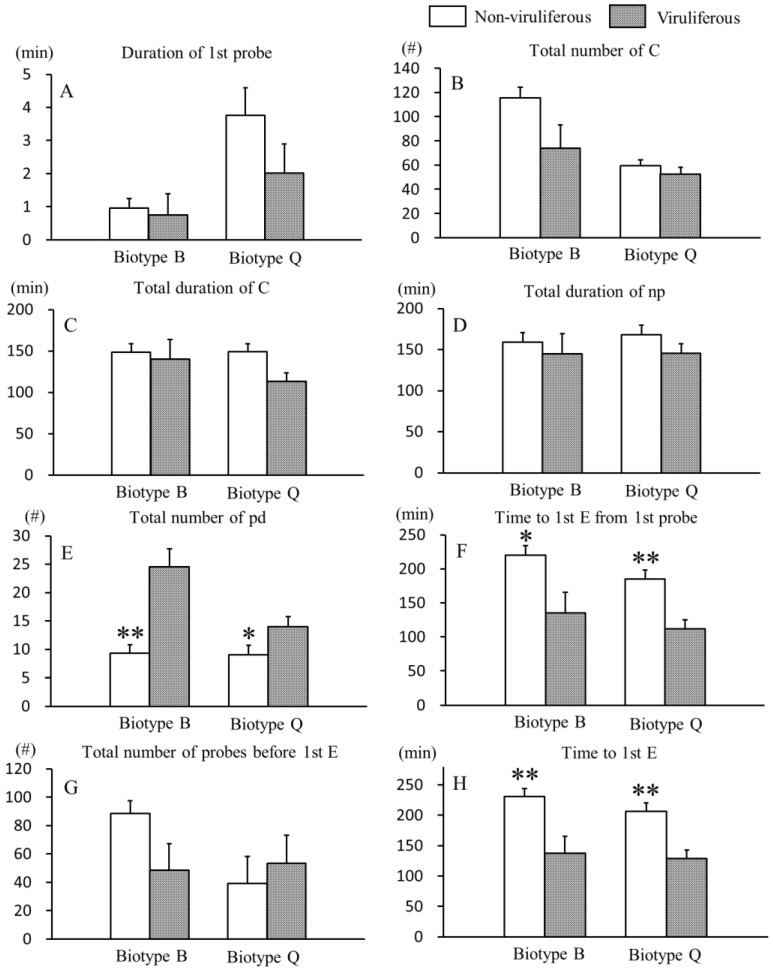
Non-phloem electrical penetration graph (EPG) variables of *B. tabaci* B and Q biotypes with cucurbit chlorotic yellows virus (CCYV). Data are means ± SE; bars with asterisk(s) (* or **) indicate a statistically significant difference between the non-viruliferous and viruliferous whiteflies of same biotype at *p* < 0.05 or *p* < 0.01 (Mann-Whitney test); EPG waveforms: C = pathway; np = non-probing/penetration; pd = potential drop (intracellular puncture); E = phloem salivary secretion and/or sap ingestion.

The effect of CCYV on the non-phloem feeding behaviors of *B. tabaci* B and Q biotypes whiteflies are shown in Figure 1. Compared to non-viruliferous whiteflies, viruliferous B and Q biotype whiteflies spent significantly shorter time to first E from first probe (for B biotype, 220.32 ± 13.98 min vs. 135.33 ± 30.06 min, *p* = 0.014; for Q biotype, 185.13 ± 13.04 min vs. 111.63 ± 13.78 min, *p* < 0.001) (Figure 1F) and time to first E (for B biotype, 230.64 ± 13.27 min vs. 137.15 ± 28.53 min, *p* = 0.004; for Q biotype, 206.49 ± 13.52 min vs. 128.49 ± 14.29 min, *p* = 0.002) (Figure 1H), and made a significantly greater total number of pd (for B biotype, 9.35 ± 1.49 vs. 24.50 ± 3.20, *p* < 0.001; for Q biotype, 9.04 ± 1.65 vs. 13.97 ± 1.74, *p* = 0.004) (Figure 1E). Other non-phloem variables, including the duration of first probe (Figure 1A), total number of C (Figure 1B), total duration of C (Figure 1C), total duration of np (Figure 1D) and total number of probes before first E (Figure 1G), did not significantly differ between viruliferous and non-viruliferous whiteflies of both biotypes.

### 3.2. Effects of CCYV on Phloem Feeding Behaviors of B. tabaci B and Q Biotypes

**Figure 2:**
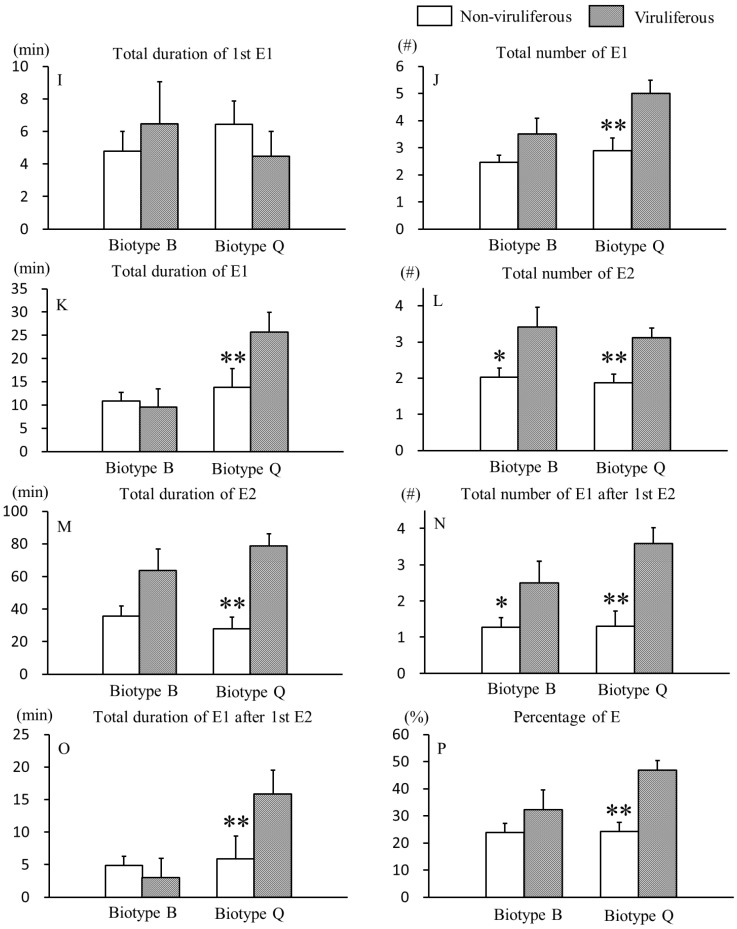
Phloem EPG variables of *B. tabaci* B and Q biotypes with CCYV. Date represent means ± SE; bars with asterisk(s) (* or **) indicate a statistically significant difference between the non-viruliferous and viruliferous whiteflies of same biotype at *p* < 0.05 or *p* < 0.01 (Mann-Whitney test); percentage of E (%) = percentage of total duration of E (E1 + E2) in the recording time. EPG waveforms: E1 = phloem salivary secretion; E2 = phloem sap ingestion.

From 8 phloem EPG variables selected (Figure 2), we found that CCYV stimulated phloem salivation and sap ingestion in both biotypes of *B. tabaci*, with stronger effects on feeding behaviors of biotype Q than those of biotype B. Viruliferous B and Q biotype whiteflies had a highly significantly greater total number of E2 (for B biotype, 2.03 ± 0.25 vs. 3.42 ± 0.54, *p* = 0.026; for Q biotype, 1.87 ± 0.25 vs. 3.12 ± 0.27, *p* < 0.001) (Figure 2L) and total number of E1 after first E2 (for B biotype, 1.26 ± 0.27 vs. 2.50 ± 0.59, *p* = 0.048; for Q biotype, 1.30 ± 0.42 vs. 3.58 ± 0.44, *p* < 0.001) (Figure 2N) than non-viruliferous whiteflies. Compared to non-viruliferous Q biotype whiteflies, viruliferous Q biotype whiteflies had a significantly greater total number of E1 (2.89 ± 0.46 vs. 5.00 ± 0.49, *p* = 0.001) (Figure 2J), total duration of E1 (13.82 ± 4.03 min vs. 25.68 ± 4.26 min, *p* = 0.001) (Figure 2K), total duration of E2 (27.93 ± 7.03 min vs. 78.68 ± 7.43 min, *p* < 0.001) (Figure 2M), total duration of E1 after first E2 (5.91 ± 3.50 min vs. 15.86 ± 3.70 min, *p* = 0.007) (Figure 2O) and percentage of E (%) (24.20 ± 3.34 vs. 46.92 ± 3.53, *p* < 0.001) (Figure 2P). However, the total duration of first E1 (Figure 2I) did not differ between viruliferous and non-viruliferous whiteflies of both biotypes.

### 3.3. Effects of CCYV on Feeding Behaviors of the Different Sexes of B. tabaci B and Q Biotypes

The effects of CCYV on feeding behaviors of the different sex of non-viruliferous and viruliferous B and Q biotypes whiteflies were shown in Table 1 and Table 2.

#### 3.3.1. Feeding Behaviors of Male Whiteflies

*Non-phloem EPG variables* (Table 1). Compared to non-viruliferous B and Q biotypes male whiteflies, viruliferous B and Q biotypes male whiteflies had significantly shorter time to first E from first probe and time to first E (Table 1, Variables F and G). Furthermore, viruliferous B and Q biotypes male whiteflies spent significantly less total duration of np (Table 1, Variable D), and viruliferous B male whiteflies had a significantly greater total number of pd (Table 1, Variable E). No significant differences were detected between non-viruliferous and viruliferous males or females in both biotypes in the duration of first probe, total number of C, total duration of C (Table 1, Variables A, B and C).

*Phloem EPG variables* (Table 2). Viruliferous B and Q biotypes male whiteflies had a significantly greater total number of E2 and total duration of E2 (Table 2, Variables L and M) than non-viruliferous male whiteflies. Furthermore, viruliferous Q biotypes male whiteflies had a significantly greater total number of E1, total duration of E1, total number of E1 after first E2, total duration of E1 after first E2, and percentage of E (Table 2, Variables J, K, N, O and P), but non-viruliferous and viruliferous B biotypes male whiteflies did not differ in these variables. Total duration of first E1 did not differ between non-viruliferous and viruliferous males in both biotypes (Table 2, Variable I).

#### 3.3.2. Feeding Behaviors of the Female Whiteflies

*Non-phloem EPG variables* (Table 1). Viruliferous Q biotype female whiteflies had significantly shorter time to first E from first probe and time to first E (Table 1, Variables F and G), and a significantly greater total number of pd (Table 1, Variable E) than non-viruliferous Q biotype female whiteflies. Moreover, viruliferous B biotype female whiteflies had significantly shorter total number of C (Table 1, Variable B) than non-viruliferous B biotype female whiteflies. Other non-phloem variables, including duration of first probe, total duration of C, total duration of np, total number of probes before first E, did not differ between non-viruliferous and viruliferous females in both biotypes (Table 1, Variables A, C, D and G).

*Phloem EPG variables* (Table 2). Viruliferous Q biotype female whiteflies had significantly shorter total duration of first E1 than non-viruliferous Q biotype female whiteflies (Table 2, Variable I). Compared to non-viruliferous Q biotyoe female whiteflies, however, viruliferous Q biotype female whiteflies had more total duration of E2 and higher percentage of E (Table 2, Variables M and P), but non-viruliferous and viruliferous B biotypes female whiteflies did not differ in these variables. Other phloem variables, including total number of E1, total duration of E1, total number of E2, total number of E1 after first E2 and total duration of E1 after first E2, did not differ between non-viruliferous and viruliferous females in both biotypes (Table 2, Variables J, K, L, N and O).

#### 3.3.3. Comparison of Feeding Behaviors of the Male and Female Whiteflies between Biotypes

*Non-phloem EPG variables* (Table 1). Non-viruliferous B biotype male and female whiteflies had a greater total number of C and total number of probes before first E than non-viruliferous Q biotype male and female whiteflies (Table 1, Variables B and G), and non-viruliferous B biotype male whiteflies had more time to first E from first probe than non-viruliferous Q biotype male whiteflies (Table 1, Variable F), while the viruliferous B and Q biotype male and female whiteflies did not differ about these variables. Furthermore, viruliferous B biotype male whiteflies had a greater total number of pd than viruliferous Q biotype male whiteflies (Table 1, Variable E).

*Phloem EPG variables* (Table 2). Compared to viruliferous male whiteflies, non-viruliferous Q biotype male whiteflies had more total duration of first E1 than non-viruliferous B biotype male whiteflies (Table 2, Variable I). Compared to non-viruliferous female whiteflies, viruliferous Q biotype female whiteflies had more percentage of E than viruliferous B biotype female whiteflies (Table 2, Variable P).

### 3.4. Interaction Effects of CCYV, Sexes and Biotypes

#### Two-Way ANOVA Analyses

Table 3 presents the two-way ANOVA statistics on feeding behaviors of the different sexes with non-viruliferous and viruliferous *B. tabaci* B or Q biotype, respectively.

(i) *EPG variables of B biotype whiteflies*. The sex of whitefly had significant effects on 3 out of 16 variables examined, i.e., the total duration of np, total number of pd and total duration of E2 (Table 3, Variables D, E and M). Whitefly viruliferous status appeared to exert significant effect on 5 of 16 variables, including total number of pd, time to first E from first probe, time to first E, total number of E2, total duration of E2, total number of E1 after first E2 (Table 3, Variables E, F, H, L and N). The interaction of the two variables exerted a significant effect on the total duration of np, total number of pd, time to first E from first probe, time to first E, total duration of E2 (Table 3, Variables D, E, F, H and M).

(ii) *EPG variables of Q biotype whiteflies*. The sex of whitefly appeared to exert significant effect on 5 out of 16 variables, i.e., the total duration of C, total duration of np, total number of E1, total number of E2, total number of E1 after first E2 (Table 3, Variables C, D, J, L and N). The viruliferous status exerted significant effects on 10 variables, including total number of pd, time to first E from first probe, time to first E, total number of E1, total duration of E1, total number of E2, total duration of E2, total number of E1 after first E2, total duration of E1 after first E2, and percentage of E (Table 3, Variables E, F, H, J, K, L, M, N, O and P). The interaction of the two variables showed a significant effect on the total number of E2, total duration of E2, and total duration of E1 after first E2 (Table 3, Variables L, M and O). It clearly indicated that the whitefly viruliferous status exerted remarkably stronger effects on whitefly feeding behaviors than the sex on cotton plants, and viruliferous status had stronger effects on Q biotype than B biotype.

### 3.5. Multivariate Analyses

Table 4 presents the multivariate statistics on feeding behaviors of the different sex with non-viruliferous and viruliferous *B. tabaci* B and Q biotypes. The biotype of whitefly had significant effects on 3 out of 16 variables, i.e., the total duration of C, total number of pd, and total number of probes before first E (Table 4, Variables B, E and G). The sex of whitefly had significant effects on 4 out of 16 variables, including the total duration of C, total duration of np, total number of pd and total duration of E2 (Table 4, Variables C, D, E and M). Whitefly viruliferous status showed significant effect on 4 non-phloem variables (the total number of pd, time to first E from first probe, total number of probes before first E, time to first E) and 5 phloem variables (total number of E1, total number of E2, total duration of E2, total number of E1 after first E2 and percentage of E) (Table 4, Variables E, F, G, H, J, L, M, N and P). Interaction of the biotype and sex exerted a significant effect on the total duration of E2 (Table 4, Variable M), and the interaction of biotype and viruliferous status showed a significant effect on the total number of pd (Table 4, Variable E), while the interaction of sex and viruliferous status appeared to exert significant effect on the total duration of np, total number of pd, total number of E2, total duration of E2 (Table 4, Variable D, E, L and M). The interaction of the biotype, sex and viruliferous status exerted a significant effect on the total number of pd (Table 4, Variable E). It clearly indicated that the whitefly viruliferous status exerted remarkably stronger effects on whitefly feeding behaviors than the sex or biotype on cotton plants.

## 4. Discussion

In this study, we found that *Cucurbit chlorotic yellows virus* (CCYV) could differentially manipulate feeding behaviors of its vector *B. tabaci* with varying degrees on sexes and biotypes. This is the first report about a semipersistly transmitted virus manipulating feeding behaviors of different sexes and biotypes of its whitefly vector. Importantly, the altered feeding behaviors of viruliferous whiteflies seemed likely to increase the rates of CCYV transmission. Viruliferous whiteflies spent more time salivating into sieve elements than did non-viruliferous whiteflies. Salivation is essential for the virus transmission [5]. More time spent in E1, and a shift toward a larger number of short feeding bouts, should maximize viral inoculation [7].

In the EPG experiments, we used cotton plants because they are excellent host plants for whiteflies and immune to CCYV. This set-up enabled us to eliminate the effects of plant-mediated indirect modifications due to virus infection [47] that could mask the direct effects of the virus on its vector. Here, we found that viruliferous whiteflies showed significantly higher feeding efficiency, and the interactions between plant viruses and their insect vectors are not neutral. The non-viruliferous B and Q biotypes whiteflies, for example, took about 1.5 times more time probing to the phloem of plants than viruliferous whiteflies (Figure 1F,H), while viruliferous whiteflies had about 1.4 times more in the total number of E2 (Figure 2L) and about 1.5 times greater total number of E1 after first E2 (Figure 2N) than non-viruliferous whiteflies. Similarly, compared to non-viruliferous whiteflies, whiteflies carrying *Tomato yellow leaf curl virus* (TYLCV, a persistently transmitted virus) took more salivating time into sieve tube elements and reached to the phloem of plants more quickly [7], and viruliferous whiteflies were more restless and had more attempted probes and salivation [8].

B and Q biotypes whiteflies of *B. tabaci* differed in several aspects of their feeding behaviors in regard to CCYV effects. Compared to non-viruliferous whiteflies, for example, viruliferous Q biotype whiteflies had a significantly greater total number of E1, total duration of E1, total duration of E2, total duration of E1 after first E2 (Figure 2J,K,M,O) than viruliferous B biotype whiteflies. The total time spent in sap ingestion (Figure 2M) was most strongly correlated with viral acquisition, and the total time spent in salivating into sieve elements (Figure 2J,K,O) was most strongly correlated with viral inoculation [5]. This agrees with the results of the previous study [7] that *B. tabaci* B and Q biotype whiteflies were affected similarly by the presence of TYLCV, but this does not mean that they are equally effective viral vectors. Our study found that Q biotype whiteflies had a stronger ability to transmit CCYV, the semipersistent virus, than B biotype whiteflies [48].

In *B. tabaci* and many other piercing-sucking insects, males are smaller in size and more active than females, and faster movements of males may facilitate virus transmission. In the coevolution of plant viruses and their insect vectors, viruses probably rely more on males than females in dispersal, so it can be presumed that feeding behaviors of males may be manipulated greater in males by viruses. In this work, we found that the feeding behaviors of different sexes of viruliferous B and Q biotypes whiteflies were manipulated to different degrees when they were carrying CCYV (Table 3). For example, viruliferous male whiteflies of both biotypes could reach to the phloem of plants more quickly (Table 1, Variables F and H) and spend more time feeding on sap (Table 2, Variable L) than viruliferous female whiteflies. Compared to non-viruliferous males or females, CCYV had shortened the difference of degree between male whiteflies of B and Q biotypes (Table 1, Variables B, F and G), and enlarged the difference of degree between females of B and Q biotypes (Table 1, Variable G; Table 2, Variable P). Ning and collaborators [49] reported that the feeding ability of TYLCV-infected whiteflies was significantly affected by the sex. CCYV-infected whitefly males and females had a greatly different performance between B and Q biotypes whiteflies. Similar result was reported in Stafford and collaborators [6] that the feeding behavior of the thrip *Frankliniella occidentalis* males carrying *Tomato spotted wilt virus* (TSWV) was modified to enhance virus transmission. Moreover, in this study, the viruliferous B biotype male whiteflies had significant more potential drops (pd), representing puncturing into the tissue cells and tasting the cytoplasm (Figure 1E; Table 1, Variable E), than non-viruliferous male whiteflies. The intracellular probing is related to the spread of non-persistent viruses [50], and the relationship between the intracellular probing and the spread of phloem-restricted semipersistent virus needs further study.

Manipulation of vector feeding behaviors by the plant virus is an evolved mechanism to facilitate virus transmission [51,52,53,54,55], but mechanism underlying this phenomenon is not clear. A number of internal and external factors, such as the age of the vector, virus quantity within the vector, disease progressing, and the growth stage of the plant, lead to the three-way complex dynamic interactions among viruses, vectors and plants [56]. Some studies had revealed the interactions between a virus and its vectors may vary with the vector and plant species, even with different strains of the same species. TSWV activates the immune system of its vector, the *F. occidentalis* [57]. Moreover, transcriptome analysis of *B. tabaci* MEAM1 putative species (Q biotype) with or without *Tomato yellow leaf curl China virus* (TYLCCNV) showed that TYLCCNV may have direct adverse effects on MEAM1, such as disturbed circulation and metabolism in the cell [58]. Wang and collaborators [59] have studied the Angiotensin-converting enzymes (ACEs) in aphid saliva finding that when ACE1 and ACE2 were simultaneously knocked down, aphid feeding was enhanced, such as aphids required less time to reach the phloem and showed longer passive ingestion. Does this suggest that virus particles binding to vector receptors can change some gene expression and thereby enhance the feeding behaviors of whiteflies or other insect vectors? In addition, whether endosymbionts of the organisms affect the tripartite interactions among viruses, vectors and plants is still barely understood [60,61]. The presence of *Hamiltonella* was involved in acquisition, retention, and transmission of TYLCV by *B. tabaci* B and Q biotypes in significant differences for TYLCV accumulation in plants exposed to the whiteflies [62]. CCYV and some other *Crinivirus* were proven to bind to receptors in the foregut within the whitefly vectors [3,48], but little information is available in molecular mechanisms underlying changes of vectors’ feeding behaviors when carrying these semipersistently transmitted viruses.

## 5. Conclusions

In conclusion, our current study confirmed that CCYV, a non-circulative phloem restricted *Crinivirus*, could manipulate the probing and feeding behavior of its vector, *B. tabaci*, in a manner that facilitates its own transmission. Interestingly, the manipulation degrees vary with sexes and the biotypes of whiteflies. We found that CCYV had more effects on feeding behaviors in Q biotype than in B biotype, by increasing duration of phloem salivation and sap ingestion. CCYV could differentially manipulate feeding behaviors of males and females in both biotype whiteflies, by the order as Q males > B males > Q females > B females, with more phloem ingestion in males than females in Q biotype and more non-phloem probing in males than females in B biotype. These results clearly indicated that the probing and feeding behaviors of *B. tabaci* B and Q biotypes changed greatly when carrying CCYV, with varying degrees with biotypes and sexes. From these EPG results of feeding behavioral changes of whitefly vectors carrying CCYV, we can presume that *B. tabaci* Q biotype plays more roles than B biotype, and males make greater contribution than females in CCVY transmission. Our studies have clear implications for understanding the behavioral mechanisms underlying a mutualistic relationship between an insect vector and a plant virus, and may help to improve management of different *B. tabaci* biotypes and the semipersistly transmitted virus (CCYV and other *Crinivirus* virus species).

## Figures and Tables

**Table 1:** EPG non-phloem variables of different sexes of non-viruliferous and viruliferous *B. tabaci* B and Q biotypes.

Variables	Biotype	Non-Viruliferous Male	Viruliferous Male	*p* ^3^ Value	Non-Viruliferous Famale	Viruliferous Famale	*p* Value
A, duration of 1st probe (min)	B	0.96 ± 0.42a ^1^	0.29 ± 1.02a ^2^	0.550	0.95 ± 0.42a	1.21 ± 0.75a	0.761
	Q	6.02 ± 1.67a	1.63 ± 1.59a	0.064	1.49 ± 0.70a	2.40 ± 0.83a	0.406
B, total number of C ^4^ (#)	B	123.67 ± 14.77a	88.00 ± 36.17a	0.373	106.84 ± 10.48a	60.17 ± 18.65a	**0.040**
	Q	55.35 ± 7.03b	56.64 ± 6.70a	0.895	63.50 ± 6.77b	49.19 ± 8.09a	0.181
C, total duration of C (min)	B	148.94 ± 16.23a	170.37 ± 39.77a	0.624	147.36 ± 14.45a	110.48 ± 25.72a	0.224
	Q	168.21 ± 13.87a	131.18 ± 13.23a	0.061	129.60 ± 12.55a	95.36 ± 15.00a	0.086
D, total duration of np (min)	B	164.86 ± 13.15a	70.41 ± 32.20a	**0.014**	153.84 ± 18.32a	218.72 ± 32.60a	0.096
	Q	157.01 ± 15.31a	107.93 ± 14.60a	**0.026**	179.66 ± 15.41a	183.13 ± 18.42a	0.886
E, total number of pd (#)	B	8.11 ± 1.76a	34.00 ± 4.30a	**<0.001**	10.58 ± 2.34a	15.00 ± 4.16a	0.364
	Q	9.65 ± 2.39a	15.18 ± 2.28b	0.102	8.43 ± 2.18a	12.76 ± 2.61a	**0.019**
F, time to 1st E from 1st probe (min)	B	246.30 ± 16.33a	89.38 ± 39.99a	**0.002**	194.33 ± 22.05a	181.27 ± 39.24a	0.774
	Q	171.49 ± 19.17b	94.76 ± 18.28a	**0.006**	198.78 ± 17.15a	128.51 ± 20.50a	**0.011**
G, total number of probes before 1st E (#)	B	103.50 ± 14.88a	46.00 ± 36.44a	0.160	73.68 ± 9.94a	51.00 ± 17.68a	0.275
	Q	33.10 ± 5.56b	24.45 ± 5.30a	0.267	45.44 ± 31.85b	82.12 ± 38.07a	0.463
H, time to 1st E (min)	B	251.85 ± 16.90a	92.13 ± 41.39a	**0.002**	209.43 ± 20.06a	182.18 ± 35.70a	0.512
	Q	198.24 ± 20.07a	111.47 ± 19.13a	**0.003**	214.74 ± 17.67a	145.51 ± 21.13a	**0.015**

^1^ Data are expressed as Mean ± SE; ^2^ Letters immediately after the mean values were derived from the comparisons of different *B. tabaci* biotypes within the same sex and infection situation. Means followed by the same letters do not differ significantly (Mann-Whitney test, *p* < 0.05); ^3^ Comparison of feeding behaviors of different sex with non-viruliferous and viruliferous *B. tabaci* B and Q biotypes (Mann-Whitney test, *p* < 0.05). ^4^ EPG waveforms: C = pathway; np = non-probing/penetration; pd = potential drop (intracellular puncture); E = phloem salivary secretion and/or sap ingestion.

**Table 2:** EPG phloem variables of different sexes of non-viruliferous and viruliferous *B. tabaci* B and Q biotypes.

Variables	Biotype	Non-Viruliferous Male	Viruliferous Male	*p* ^3^ Value	Non-Viruliferous Famale	Viruliferous Famale	*p* Value
I, total duration of 1st E1 (min)	B	2.88 ± 0.89a ^1^	6.93 ± 2.19a ^2^	0.103	6.72 ± 2.12a	6.03 ± 3.78a	0.875
	Q	5.84 ± 2.65b	5.03 ± 2.53a	0.826	7.05 ± 1.45a	3.94 ± 1.73a	**0.025**
J, total number of E1 ^5^ (#)	B	2.33 ± 0.40a	4.00 ± 0.97a	0.129	2.58 ± 0.37a	3.00 ± 0.65a	0.579
	Q	3.05 ± 0.65a	6.09 ± 0.62a	**0.002**	2.73 ± 0.62a	3.90 ± 0.74a	0.233
K, total duration of E1 (min)	B	9.18 ± 2.46a	10.89 ± 6.02a	0.795	12.49 ± 2.68a	8.13 ± 4.77a	0.434
	Q	11.65 ± 3.66a	23.78 ± 3.49a	**0.021**	16.00 ± 6.32a	27.58 ± 7.55a	0.245
L, total number of E2 (#)	B	2.00 ± 0.35a	4.00 ± 0.86a	**0.044**	2.05 ± 0.36a	2.83 ± 0.65a	0.304
	Q	1.80 ± 0.43a	4.09 ± 0.41a	**<0.001**	1.93 ± 0.29a	2.14 ± 0.35a	0.646
M, total duration of E2 (min)	B	35.55 ± 8.13a	106.03 ± 19.90a	**0.004**	36.01 ± 9.00a	21.22 ± 16.02a	0.429
	Q	22.13 ± 11.89a	95.49 ± 11.33a	**<0.001**	33.72 ± 8.18a	61.87 ± 9.77a	**0.032**
N, total number of E1 after 1st E2 (#)	B	1.06 ± 0.40a	3.00 ± 0.98a	0.082	1.47 ± 0.37a	2.00 ± 0.66a	0.496
	Q	1.40 ± 0.60a	4.55 ± 0.57a	**<0.001**	1.20 ± 0.56a	2.62 ± 0.67a	0.110
O, total duration of E1 after 1st E2 (min)	B	4.45 ± 1.78a	3.96 ± 4.37a	0.918	5.30 ± 2.06a	2.10 ± 3.66a	0.455
	Q	4.68 ± 2.60a	15.35 ± 2.48a	**0.001**	7.14 ± 5.65a	16.36 ± 6.75a	0.300
P, percentage of E (%) ^4^	B	24.58 ± 5.24a	41.15 ± 12.84a	0.247	22.99 ± 4.44a	23.29 ± 7.90a	0.891
	Q	18.05 ± 4.78a	46.63 ± 4.56a	**<0.001**	30.35 ± 4.47a	47.21 ± 5.34b	**0.019**

^1^ Data are expressed as Mean ± SE; ^2^ Letters immediately after the mean values were derived from the comparisons of different *B. tabaci* biotypes within the same sex and infection situation. Means followed by the same letters do not differ significantly (Mann-Whitney test, *p* < 0.05); ^3^ Comparison of feeding behaviors of different sex with non-viruliferous and viruliferous *B. tabaci* B and Q biotypes (Mann-Whitney test, *p* < 0.05); ^4^ percentage of E (%) = percentage of total duration of E (E1 + E2) in the recording time; ^5^ EPG waveforms: E1 = phloem salivary secretion; E2 = phloem sap ingestion.

**Table 3:** Interaction analysis of EPG variables among sexes, viruliferous status and biotypes by two-way analysis of variance (ANOVA).

Variables	*B. tabaci* B (*p* Value) ^1^			*B. tabaci* Q (*p* Value)		
	Sex	Virus	Sex * Virus	Sex	Virus	Sex * Virus
***Non-phloem variables***						
A, duration of 1st probe (min)	0.522	0.776	0.511	0.447	0.153	0.111
B, total number of C ^3^ (#)	0.267	0.057	0.533	0.730	0.373	0.237
C, total duration of C (min)	0.237	0.764	0.262	**0.024**	0.127	0.983
D, total duration of np (min)	**0.016**	0.591	**0.006**	**0.003**	0.166	0.111
E, total number of pd (#)	**0.024**	**<0.001**	**0.004**	0.45	**0.043**	0.802
F, time to 1st E from 1st probe (min)	0.550	**0.014**	**0.036**	0.085	**<0.001**	0.906
G, total number of probes before 1st E (#)	0.747	0.060	0.580	0.160	0.082	0.396
H, Time to 1st E (min)	0.453	**0.005**	**0.041**	0.202	**<0.001**	0.657
***Phloem variables***						
I, total duration of 1st E1 (min)	0.721	0.558	0.135	0.978	0.347	0.580
J, total number of E1 (#)	0.558	0.110	0.335	**0.006**	**0.001**	0.084
K, total duration of E1 (min)	0.950	0.762	0.488	0.484	**0.001**	0.146
L, total number of E2 (#)	0.361	**0.026**	0.246	**0.019**	**<0.001**	**0.006**
M, total duration of E2 (min)	**0.006**	0.061	**0.005**	0.989	**<0.001**	**0.003**
N, total number of E1 after 1st E2 (#)	0.655	**0.049**	0.279	**0.031**	**0.001**	0.094
O, total duration of E1 after 1st E2 (min)	0.493	0.575	0.351	0.116	**0.007**	**0.049**
P, percentage of E (%) ^2^	0.235	0.302	0.319	0.189	**<0.001**	0.231

^1^ *p* values calculated using two-way ANOVA with main effects of sex (male and female), infection (non-viruliferous and viruliferous), and their interaction; *p* values in boldface are significant at *p* < 0.05; ^2^ percentage of E (%) = percentage of total duration of E (E1 + E2) in the recording time; ^3^ EPG waveforms: C = pathway; np = non-probing/penetration; pd = potential drop (intracellular puncture); E1 = phloem salivary secretion; E2 = phloem sap ingestion.

**Table 4:** Interaction analysis of EPG variables among sexes, viruliferous status and biotypes of *B. tabaci* by multivariate statistics.

Variables	*p* Value ^1^						
	Biotype	Sex	Virus	Biotype * Sex	Biotype * Virus	Sex * Virus	Biotype * Sex * Virus
Non-phloem variables							
A, duration of 1st probe (min)	0.167	0.932	0.796	0.511	0.634	0.972	0.111
B, total number of C ^3^ (#)	**<0.001**	0.271	0.193	0.443	0.205	0.272	0.957
C, total duration of C (min)	0.367	**0.021**	0.139	0.824	0.340	0.342	0.296
D, total duration of np (min)	0.766	**0.001**	0.262	0.555	0.810	**0.002**	0.112
E, total number of pd (#)	**0.024**	**0.035**	**<0.001**	0.177	**0.033**	**0.018**	**0.035**
F, time to 1st E from 1st probe (min)	0.136	0.201	**<0.001**	0.788	0.770	0.058	0.083
G, total number of probes before 1st E (#)	**0.001**	0.687	**0.060**	0.340	0.837	0.946	0.377
H, time to 1st E (min)	0.411	0.219	**<0.001**	0.971	0.697	0.061	0.151
Phloem variables							
I, total duration of 1st E1 (min)	0.932	0.707	0.945	0.728	0.369	0.386	0.764
J, total number of E1 (#)	0.120	0.190	**0.012**	0.480	0.391	0.210	0.801
K, total duration of E1 (min)	0.072	0.681	0.321	0.720	0.215	0.755	0.794
L, total number of E2 (#)	0.538	0.051	**0.001**	0.638	0.851	**0.028**	0.563
M, total duration of E2 (min)	0.976	**0.041**	**0.002**	**0.042**	0.330	**0.001**	0.634
N, total number of E1 after 1st E2 (#)	0.462	0.199	**0.001**	0.359	0.710	0.132	0.848
O, total duration of E1 after 1st E2 (min)	0.190	0.187	0.128	0.797	0.213	0.087	0.788
P, percentage of E (%) ^2^	0.130	0.741	**0.002**	0.106	0.152	0.161	0.819

*p* values calculated using multivariate analysis with main effects of biotype (B and Q), sex (male and female), infection (non-viruliferous and viruliferous), and their interaction; *p* values in boldface are significant at *p* < 0.05; ^2^ percentage of E (%) = percentage of total duration of E (E1 + E2) in the recording time. ^3^ EPG waveforms: C = pathway; np = non-probing/penetration; pd = potential drop (intracellular puncture); E1 = phloem salivary secretion; E2 = phloem sap ingestion.
